# Improvement in the Analytical Capabilities of LA-ICP-MS for High Spatial Resolution U-Pb Dating of Zircon Using Mixed-Gas Plasma

**DOI:** 10.1155/2020/1819639

**Published:** 2020-05-11

**Authors:** Liu Shenghua, Wang Xin, Shi Huixia, Zhang Hui, Liu Bingbing

**Affiliations:** ^1^School of Earth Sciences and Resources, China University of Geosciences (Beijing), Beijing 100083, China; ^2^Key Laboratory of Groundwater Science and Engineering, Ministry of Natural Resources, Institute of Hydrology and Environmental Geology, CAGS, Shijiazhuang 050061, China; ^3^The First Geological Exploration Institute of Henan Bureau of Geo-Exploration and Mineral Development, Zhengzhou 450000, China

## Abstract

In this work, a novel method for high spatial resolution U-Pb dating of zircon by laser ablation inductively coupled plasma mass spectrometry (LA-ICP-MS) at 10–16 *μ*m spot diameter has been proposed. This was achieved by introducing 2% (*v*/*v*) water-ethanol vapours into ICP in combination with the shielded torch system to increase the sensitivity and suppress the isotopic fractionation effect. Precise and accurate concordant U-Pb ages for Plešovice, GJ-1, and 91500 zircons were obtained using the proposed method, and the results agreed well with the isotope dilution-thermal ionization mass spectrometry (ID-TIMS) and LA-ICP-MS within 2*σ*, except for the ages obtained using dry plasma at 10 *μ*m spot diameter. Additionally, the effects of plasma condition (dry plasma or 2% (*v*/*v*) ethanol plasma) and spot size (10, 16, 24, or 32 *μ*m) on precision (RSD), accuracy (RE), and uncertainty (2*σ*) of the ^206^Pb/^238^U ages have been studied. The results indicated that increasing the spot diameter and introducing 2% (*v*/*v*) water-ethanol vapours into ICP significantly improved the precision, accuracy, and uncertainty for small spot diameters (10 and 16 *μ*m), while it exhibited little influence for intermediate spot diameters (24 and 32 *μ*m). Furthermore, the effects of spot size and plasma condition on the precision, accuracy, and uncertainty strongly depended on the sensitivity of analytes.

## 1. Introduction

Fine elemental and isotopic composition information of minerals at microscale are beneficial to decode the crystallization environment, the experienced geochemical process, and the history of geological body evolution. Due to the advantages of minimal sample preparation, high throughput, high spatial resolution, and small sample requirements, LA-ICP-MS has become the preferred technology for the microscale analysis in solid samples, such as mineral zonation dating and elemental and isotopic mapping [1–4]. Although the capability of LA-ICP-MS was significantly improved by technological progress in laser ablation system and ICP-MS instrument, high spatial resolution analysis by LA-ICP-MS was yet hampered by low sensitivity with an inferior precision and perceptible isotopic fractionation. Therefore, a novel method having higher analytical sensitivity, precision, and accuracy for high spatial resolution analysis using LA-ICP-MS is desired.

In quest of enhancing the analytical sensitivity of LA-ICP-MS, high laser ablation repetition rate and large spot diameter are often employed as a common LA-ICP-MS strategy. However, such a strategy increases the consumption of sample and the chance of elemental fractionation during laser ablation. More important limitation of large spot diameter is not considered suitable for high spatial resolution analysis, as the data usually represents a mixed information in 32 *μ*m or even on a larger scale. Alternatively, the addition of small amounts of a few active matrixes, such as helium [5], nitrogen [6, 7], hydrogen [8], methane [9], ethanol [10, 11], and water vapours [12], into traditional argon plasma has been studied for improving signal intensity, suppressing spectral interferences, and reducing matrix effect in many publications. Günther and Heinrich [5] reported that, in addition to greatly reducing the background intensity across the entire mass range, a high flux of helium yielded a 2-3-fold enhancement in the sensitivity, thus resulting in an order of magnitude improvement in the detection limits for most elements. Furthermore, the addition of N_2_ (5–10 ml·min^−1^) enhanced the sensitivity of most elements by a factor of 2-3, while it significantly reduced the production of oxides and hydrides by 10- and 3-fold, respectively. Additionally, the nonspectral interference effect was also reduced, although the doubly charged ion ratio and the background signals of ions (mass 29, 34, 42, 51, 52, and 55) were enhanced [6]. Guillong and Heinrich [8] reported that the sensitivity for most elements was enhanced by 2–4-fold and by 5–7-fold for Be, P, As, Pt, and Au by introducing small quantity of hydrogen into the dry aerosol generated by the laser ablation. Many studies have investigated the effects of organic solvents or gas on the sensitivity of analyte and the level of interference. For example, Fliegel et al. [9] observed a significantly enhanced sensitivity (by a factor of at least 2) for most elements investigated by adding 0.6–1.4 ml·min^−1^ methane, whereas when methanol-water mixture was added, the sensitivity increased by a factor of 20. With an introduction of ethanol and/or water vapours in a small quantity in combination with a shielded torch into LA-ICP-MS system, a signal enhancement of around 1.5–3.0-fold was achieved for 60 elements, whereas, except for the hydride ratio, the production of oxides and doubly charged ions was significantly reduced [11]. As matrix modifier, organic solvents are widely used in solution nebulization inductively coupled plasma mass spectrometry (SN-ICP-MS). However, only a handful studies have been devoted to applications of mixed-gas plasma LA-ICP-MS with the introduction of organic solvents.

The signal enhancement and low-level interference effect of mixed-gas plasma inspired us to study the probability of mixed-gas plasma application in high spatial resolution U-Pb dating. According to our prior study, to maximize analytical sensitivities of Pb, Th, and U while keeping minimum spectral interferences, herein 2% (*v*/*v*) ethanol solution was utilized to produce water-ethanol vapours generated ethanol mixed-gas plasma (*viz.,* ethanol plasma). The aim of this study is to develop a novel high spatial resolution analysis method with LA-ICP-MS by ethanol mixed-gas plasma in combination with a shielded torch. Compared to the dry plasma (without any addition of water/ethanol vapours), the effect of ethanol plasma on the precision and accuracy of determined ^206^Pb/^238^U ages at small (10 and 16 *μ*m) and intermediate (24 and 32 *μ*m) spot diameter was discussed. The new method was validated by determining the U-Pb ages of SRMs zircon Plešovice, GJ-1, and 91500.

## 2. Materials and Methods

### 2.1. Experimental Setup

The study was performed on an Agilent 7500a ICP-MS (Agilent Technology, Tokyo, Japan) coupled with 193 nm ArF-Excimer laser ablation system (GeoLas 2005, Lambda Physik, Göttingen, Germany) at the State Key Laboratory of Geological Processes and Mineral Resources (GPMR), China University of Geosciences, Wuhan, China. To avoid the transient signal with a large fluctuation at low sensitivity, a “wire” signal-smoothing device developed by Hu et al. [13] was mounted between ablation cell and ICP torch. Helium (approx. 0.5 l·min^−1^) used as the carrier gas flowed through the ablation cell, after signal-smoothing device mixed with makeup gas flow via a T-connector. Before starting experiments, the instrument was kept running for at least 30 minutes to warm up. The typical sampling depth was fixed at 5 mm in routine operation. SRM NIST 610 (NIST, Gaithersburg, USA) was ablated (at 32 *μ*m, 6 Hz in single spot mode) to optimize the position of ICP torch (*x*/*y*), the makeup gas flow rate, and the ion lenses setup for maximizing the ^238^U^+^ signal while minimizing oxide ratio (^248^ThO^+^/^232^Th^+^ < 0.5%) and elemental fractionation (^238^U^+^/^232^Th^+^ ≈ 1). The details of LA-ICP-MS operating parameters are summarized in Table 1, while the instrument tuning procedures can be found elsewhere in the literatures [6, 11, 14].

**Figure 1:**
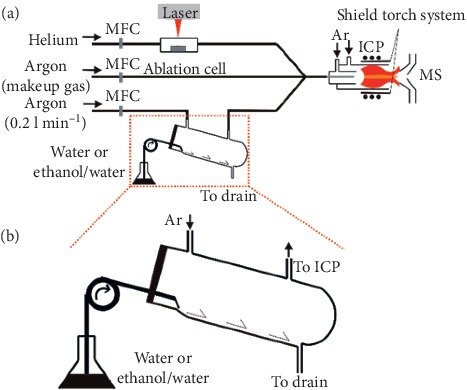
(a) Schematic of the water/ethanol vapours introducing system. (b) Custom-made introducer with the length, width, and depth of 10 cm, 3 mm, and 1 mm. MFC denotes mass flow controller.

Figure 1(a) shows the schematic of the LA-IC-MS configurations for water/ethanol vapours introduction. The introducer described in [11] was used to directly introduce water/ethanol vapours along with an argon flow (approx. 0.2 l·min^−1^) into dry aerosol which was generated by the laser ablation. In order to add water/ethanol vapours more stable for a long-term, the introducer was modified by carving a drainage groove (length of 10 cm; width of 3 mm; depth of 1 mm) on the internal surface (Figure 1(b)).

### 2.2. Reagents and Certified Reference Materials

Ultrapure water having the resistivity of 18 MΩ·cm^−1^ was produced using a Milli-Q water purification system (Millipore, Billerica, MA, USA) and was used to prepare the aqueous water-ethanol mixture. Absolute ethanol (analytical reagent; ≥99.7%, Tianjin Beilian Fine Chemicals Development Co., Ltd., Tianjin, China) was diluted to 2% (*v*/*v*). Standard reference material NIST 610 (silicate glass) was used as elemental calibration external standard. Zircon 91500 was used as U-Th-Pb isotope fractionation calibration external standard. Younger zircon standards (SRM Plešovice and GJ-1) and relative older zircon standard (SRM 91500) were used as unknow samples to validate the method. The details of Plešovice, GJ-1, and 91500 zircon crystal are specified as follows:

- *Plešovice*. Zircon comes from a high-temperature potassic granulite in the southern Bohemian Massif, Czech Republic. Although Plešovice zircon is inhomogeneous with respect to trace elements, it is homogeneous and concordant in U-Pb age. A concordant U-Pb age of Plešovice zircon determined by ID-TIMS was 338 ± 1 Ma (lower intercept U-Pb age) and 337.13 ± 0.37 Ma (the weighted mean ^206^Pb/^238^U age) presented by Aftalion et al. [15] and Sláma et al. [16], respectively. Weighted mean ^206^Pb/^238^U age reported by Frei et al. using LA-SF-ICP-MS was 338 ± 1 Ma [17].
- *GJ-1*. The standard employed in this work was a large (1 cm), gem quality, pink zircon. A bag of similar pink (and yellow) zircon was acquired from a Sydney gem dealer. The ID-TIMS ^206^Pb/^238^U age for this zircon was 599.8 ± 4.5 Ma (2*σ*, mean of the apparent ages) [18]. Based upon the U-Pb method, the LA-ICP-MS ^206^Pb/^238^U ages for this zircon were 604.6 ± 2.9 Ma (2*σ*) [19] and 603.2 ± 2.4 Ma (2*σ*) [20], which were obtained with the ablation spot size of 20 *μ*m.
- *91500*. Zircon 91500 is one of most widely applied zircon standards for U-Pb dating and Hf isotope analysis. This standard is relatively homogeneous in terms of U-Pb isotopic composition, although overall U and Pb concentrations are low [19, 21]. Its ID-TIMS age has been determined to be 1062.4 ± 0.8 Ma (2*σ*) for ^207^Pb/^206^Pb and 1065.4 ± 0.6 Ma (2*σ*) for ^206^Pb/^238^U, respectively [21]. The LA-ICP-MS ^206^Pb/^238^U age for this zircon was 1061.8 ± 5.3 Ma (2*σ*) by Belousova et al. using U-Pb method [19] and 1064.4 ± 4.8 Ma (2*σ*) by Liu et al. with ablation spot size 20 *μ*m [20].

### 2.3. Data Acquisition and Reduction

All data were acquired in the static (single spot) ablation mode. The plasma was stabilized for 5 minutes with/without the introduction of 2% (*v*/*v*) water-ethanol vapours. Each analysis incorporated a background acquisition for approximately 20–30 s (gas blank) followed by 50 s of data acquisition from the sample. SRMs Plešovice, GJ-1, and 91500, used as the unknown samples, were determined under different experimental conditions. The laser ablation repetition rate was set at 3 Hz. The experimental conditions consisted of the permutations with 2 different plasma conditions (dry plasma and 2% (*v*/*v*) ethanol plasma) at 4 different ablation spot sizes (10, 16, 24, and 32 *μ*m).

The Agilent ChemStation was used for the acquisition of each individual analysis. Offline selection, integration of the background and analyte signals, and time-drift correction and quantitative calibration for trace elements' analysis and U-Pb dating were performed using ICPMSDataCal [22, 23]. Zircon 91500 was used as the external standard for U-Pb dating and analysed twice after every five analyses. Time-dependent drifts of U-Th-Pb isotopic ratios were corrected using linear interpolation (with time) for every five analyses according to the variations of 91500 (i.e., 2 zircon 91500 + 5 samples + 2 zircon 91500) [23]. Preferred U-Th-Pb isotopic ratios were used for 91500 and were obtained from [21]. The uncertainty of preferred values for the external standard 91500 was propagated to the ultimate results of the samples. Concordia diagrams and the weighted mean calculations were undertaken using Isoplot/Ex_ver3 [24]. The precision (relative standard deviation; RSD) was calculated according to the equation ($\text{RSD}=\left(\sigma \;\text{meas}.|/|\bar{X}\;\text{meas}.\right)\times 100\%$), and the deviation in various measurements was represented. The uncertainty (2*σ*) was obtained using the software ICPMSDataCal 9.0, and the average uncertainty represented the deviation of repeatedly scanning in one certain measurement. The accuracy (relative error; RE) was calculated using the equation ($\text{RE}=\left(\bar{X}\;\text{meas}.-X\;\text{ref}./X\;\text{ref}.\right)\times 100\%$), and the error between the measured value and the reference value was represented.

## 3. Results and Discussion

### 3.1. Small Spot Diameter (10 and 16 *μ*m)

**Figure 2:**
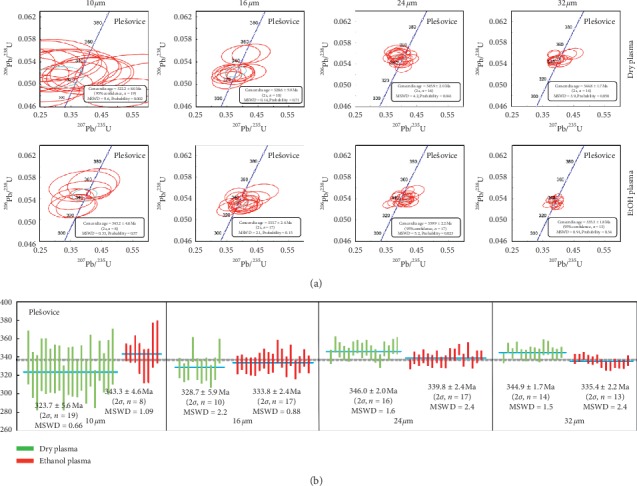
(a) U-Pb concordia diagrams and ^206^Pb/^238^U ages of zircon SRM Plešovice determined under different experimental conditions. The blue solid line represents the weighted mean ^206^Pb/^238^U ages, while the dotted line represents the reference ^206^Pb/^238^U age of SRM Plešovice obtained from [16]. Uncertainties are represented by 2*σ*, while MSWD is the mean square of weighted deviations. Experimental conditions: (1) plasma conditions (dry plasma or 2% ethanol plasma), (2) spot sizes (10, 16, 24, or 32 *μ*m). The concordance was lower than 90%, when dry plasma was used at the spot size of 10 *μ*m.

**Figure 3:**
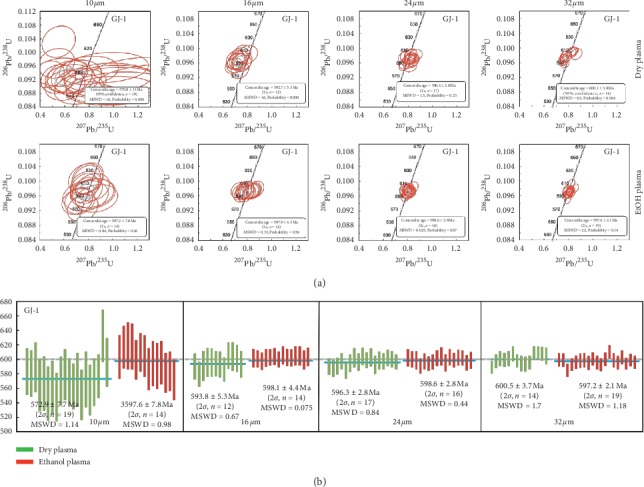
(a) U-Pb concordia diagrams and ^206^Pb/^238^U ages of zircon SRM GJ-1 determined under different experimental conditions. The blue solid line represents the weighted mean ^206^Pb/^238^U ages, while the dotted line represents the reference ^206^Pb/^238^U age of SRM GJ-1 obtained from [18]. Uncertainties are represented by 2*σ*, while MSWD is the mean square of weighted deviations. Experimental conditions: (1) plasma conditions (dry plasma or 2% ethanol plasma), (2) spot sizes (10, 16, 24, or 32 *μ*m). The concordance was lower than 90%, when dry plasma was used at the spot size of 10 *μ*m.

U-Pb ages of Plešovice and GJ-1 zircons were measured under dry plasma/ethanol plasma at small spot diameters (10 and 16 *μ*m). The concordia diagrams and the weighted mean ^206^Pb/^238^U age histograms for Plešovice and GJ-1 zircons are shown in Figures 2 and 3, respectively. The weighted mean ^206^Pb/^238^U ages and the corresponding calculated data under different experimental conditions are provided in Table 2. All single measurements of U-Pb age for these two zircons under both dry plasma and ethanol plasma at 16 *μ*m are near-concordant (concordance ≥95%). For ultrasmall spot diameter 10 *μ*m, it behaved as same as those of 16 *μ*m when measured under ethanol plasma condition. However, the results showed that single analysis was poor under dry plasma compared to ethanol plasma, under which the concordance was less than 90% and far away from the concord line in Figures 2(b) and 3(b). On the basis of these discordant ages, it is clear that the dating of zircon under dry plasma at ultrasmall spot size 10 *μ*m could not be achieved. However, the measured ages of both zircons were consistent with those obtained using the ID-TIMS and LA-ICP-MS under ethanol plasma condition and were found to be lying within 2*σ* under other experimental conditions.

The results presented in Table 2 showed that the weighted mean ^206^Pb/^238^U age (2*σ*) of Plešovice zircon was 328.7 ± 5.9 Ma under dry plasma at 16 *μ*m, while the values under ethanol plasma condition were slightly older (333.8 ± 2.4 Ma) and had lower uncertainty. The calculated relative error (RE) was 2.9% under dry plasma at 16 *μ*m, which was based upon the reference age of 337.1 ± 0.4 Ma [16]. In contrast, the relative errors were significantly low for values obtained under ethanol plasma condition (1.0%). When compared with the short-term precision (RSD) under dry plasma condition at 16 *μ*m (2.9%), the values decreased to 1.5% under ethanol plasma condition.

The weighted mean ^206^Pb/^238^U ages (2*σ*) of GJ-1 zircon were similar to each other under both the dry plasma and ethanol plasma conditions at small spot diameters. When the values obtained under both dry plasma (593.8 ± 5.3 Ma) and ethanol plasma (598.1 ± 4.4 Ma) at 16 *μ*m were compared, it was found that the value under ethanol plasma had a lower 2*σ* value. The calculated relative errors (RE) were 1.0% under dry plasma at 16 *μ*m and 0.3% under ethanol plasma conditions, whereas the reference age of 599.8 ± 4.5 Ma was obtained from literature [18].  Figure 3 showed that the data was more precise under ethanol plasma condition than that obtained under the dry plasma condition. For example, at 16 *μ*m, the corresponding short-term precision (RSD) decreased from 1.2% under dry plasma to 0.7% under ethanol plasma condition.

**Figure 4:**
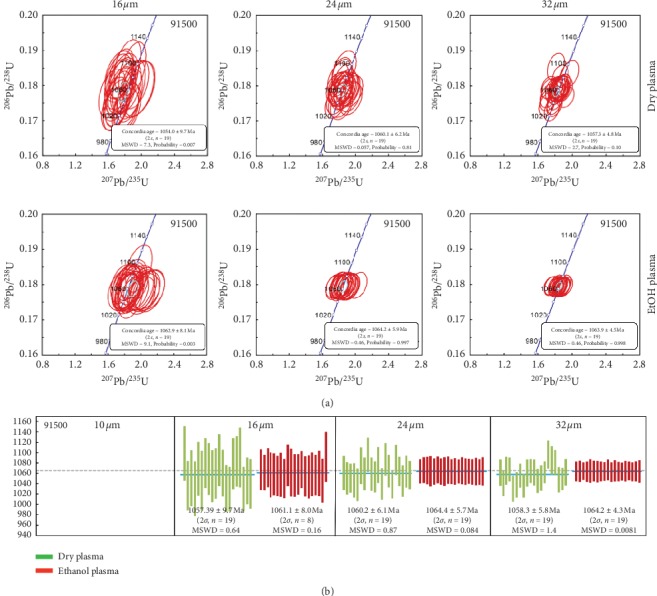
(a) U-Pb concordia diagrams and ^206^Pb/^238^U ages of zircon SRM 91500 determined under different experimental conditions. The blue solid line represents the weighted mean ^206^Pb/^238^U ages, while the dotted line represents the reference ^206^Pb/^238^U age of SRM 91500 obtained from [21]. Uncertainties are represented by 2*σ*, while MSWD is the mean square of weighted deviations. Experimental conditions: (1) plasma conditions (dry plasma or 2% ethanol plasma), (2) spot sizes (16, 24, or 32 *μ*m). The reason for the missing data of SRM 91500 at the spot diameter of 10 *μ*m is feeble signal of U and Th resulting error ages.

Due to the low U concentration (80 ± 8 ppm) and the relative older age (implying low parent isotopes being) of SRM 91500 zircon, the signal of U and Th measured is so feeble at the spot diameter of 10 *μ*m that the ages we got are error. Therefore, the SRM 91500 was only measured at 16 *μ*m rather than 10 *μ*m. The results are plotted in Figure 4 and listed in Table 2. The concordance for most of measurements is over 90% at 16 *μ*m regardless what plasma condition used. The weighted mean ^206^Pb/^238^U age (2*σ*) is 1057.9 ± 9.7 Ma and 1061 ± 8.0 Ma under dry plasma and ethanol plasma, respectively. Both of them are indistinguishable from the reference value measured by ID-TIMS (1065.4 ± 0.6 Ma) [21] and LA-ICP-MS (1061.8 ± 5.3 Ma or 1064.4 ± 4.8 Ma) [19, 20] within 2*σ*. However, the scatteration of individual measurements are narrowed from 1.8% under dry plasma to 0.8% under ethanol plasma. The RE and 2*σ* were moderately improved from 0.7%, 24.8 Ma under dry plasma to 0.4%, 20.3 Ma under ethanol plasma, respectively. By comparing these parameters under two plasma conditions, it indicated that the ethanol plasma obviously improved the capacity of LA-ICP-MS at 16 *μ*m.

### 3.2. Intermediate Spot Diameter (24 and 32 *μ*m)

U-Pb ages of Plešovice and GJ-1 zircon were determined under dry plasma/ethanol plasma at intermediate spot diameter (24 and 32 *μ*m). As can be seen from the concordia diagrams (Figures 2 and 3), it was obvious that all the single measurements yielded a concordia age under both plasma conditions (dry plasma and ethanol plasma) at intermediate spot diameter and were distributed close to the concord line. Undoubtedly, the measured ages of both zircons were in accordance with the published data by ID-TIMS and LA-ICP-MS within 2*σ*.

The weighted mean ^206^Pb/^238^U ages (2*σ*) of Plešovice zircon at 24 and 32 *μ*m were 346.0 ± 2.0 Ma and 344.9 ± 1.7 Ma under dry plasma condition, while the corresponding values under ethanol plasma condition were 339.0 ± 2.4 Ma and 335.4 ± 2.2 Ma, respectively, with keeping the same precision and uncertainty. The calculated relative errors were 2.6% and 2.8% under dry plasma condition, while the reference value 337.1 ± 0.4 Ma was used from literature [16]. From the relative error perspective, it is worth noticing that the ethanol plasma condition yielded an improved performance (0.6% and 0.5%). The change in values (trend) of GJ-1 and 91500 zircons was found to be similar to that of Plešovice zircon.

### 3.3. Effects of Plasma Condition and Spot Diameter

**Figure 5:**
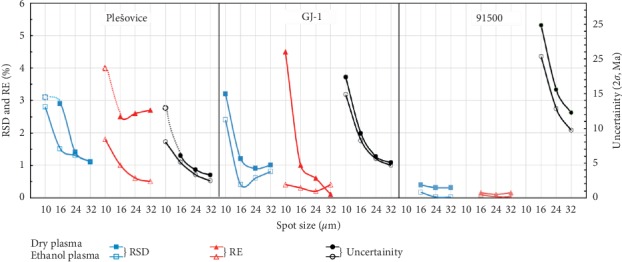
Effects of plasma condition and ablation spot diameter on RSD, RE, and uncertainty for different zircon SRM (Plešovice and GJ-1). Experimental conditions: (1) plasma conditions (dry plasma or 2% ethanol plasma), (2) spot sizes (10, 16, 24, or 32 *μ*m). It should be noted that the concordance of SRM Plešovice zircon was lower than 90%, when dry plasma was used at the spot size of 10 *μ*m, and is shown using the dotted line. The reason for the missing data of SRM 91500 at the spot diameter of 10 *μ*m is feeble signal of U and Th resulting error ages.

The effects of plasma condition and spot diameter on U-Pb age, precision, and accuracy are shown in Figure 5. Compared to dry plasma at small spot diameters, the results for short-term precision (RSD), accuracy (RE), mean uncertainty (2*σ*), and single measurement uncertainty under ethanol plasma were significantly improved. However, at intermediate spot diameter, the U-Pb ages were concordant and identical within 2*σ*, no matter what plasma condition it is. These values obtained under dry plasma condition were statistically indistinguishable from those of ethanol plasma at intermediate spot diameter. This is in contrast to results obtained at small spot diameter condition. The results reported suggested that the ethanol plasma in combination with shielded torch improved the capability of LA-ICP-MS for high spatial resolution (10–16 *μ*m) U-Pb dating of zircon. However, the ethanol plasma exhibited limited influence on the LA-ICP-MS analysis at intermediate spot diameter. With increasing in spot diameter (from 10 *μ*m to 32 *μ*m), the RSD, RE, and uncertainty values significantly enhanced, especially for the small spot diameter and that is too regardless of the type of plasma used. It is known that increasing the spot diameter (as the increase in substance load into ICP) or introducing the ethanol-water vapours (as the signal enhancement effect) [11] could improve the sensitivity for U, Th, and Pb. Meanwhile, the relationship between the uncertainty and the precision of isotopic ratio with the sensitivity of analyte could be depicted using a “hyperbolic-like” curve [25]. Based upon above discussion, it is suggested that the RSD, uncertainty, and accuracy could be improved by enhancing the analyte signal. According to the “hyperbolic-like” curve, it is worth noticing that enhancing the analyte signal to improve RSD, uncertainty, and accuracy is an efficient method when the initial sensitivity is below the inflection point. However, when the initial sensitivity lies beyond the inflection point, then enhancing analyte signal plays imperceptible role in the improvement of RSD, uncertainty, and accuracy. We quoted the above-mentioned argument as the reason for why ethanol plasma has a significant effect for small spot diameter, however limited influence for intermediate spot diameter.

It is also worth noticing that the concordance was lower than 90% under dry plasma, whereas ≥90% under ethanol plasma at the same ablation spot size of 10 *μ*m. On the one hand, the daughter Pb isotopes signals measured under dry plasma were extremely low, especially at ultrasmall spot diameter 10 *μ*m. The difference of Pb isotopes signal between SRM Plešovice and 91500 was pronounced; consequently, remarkable mass discrimination effect among Pb isotopes was observed in Plešovice rather than in 91500 [26]. It is inappropriate to calculate U-Th-Pb isotopic ratios of Plešovice zircon using external standard 91500 at ultrasmall spot diameter 10 *μ*m under dry plasma; otherwise perceptible fractionation among Pb isotopes will be determined. On the other hand, the down-hole fractionation effect was reported to be more obvious as increasing depth/diameter ratio due to ablation spot size decrease [26]. Therefore, the significant down-hole fractionation effect accelerates the fractionation between different Pb isotopes. As a result of both or either of these two factors, inconsistent Pb isotope fractionation may contribute to the discordant ages at 10 *μ*m under dry plasma condition. However, ethanol plasma (mixed-gas plasma) was proved to not only enhance the U-Th-Pb signals by a factor of about 2-3 [9, 11], but also reduce the isotopic fractionation [27]. There is no doubt that the ethanol plasma is familiar for obtaining concordant U-Pb age for young zircon even under ultrasmall spot diameter. But for extremely young or old zircon with very low daughter or parent isotope composition, such as SRM Penglai and 91500 zircons, the proposed method in this study may be invalid under ultrasmall spot diameter. Therefore, the U-Pb dating and the determination of trace elemental concentrations in extremely young or old zircon with high spatial resolution need further more research.

## 4. Conclusions

In this work, zircon U-Pb ages were measured using LA-ICP-MS at high spatial resolution (small spot sizes of 10 and 16 *μ*m) by introducing 2% water/ethanol vapours in ICP with shielded torch assistance. The mean weighted ^206^Pb/^238^U ages of Plešovice, GJ-1, and 91500 zircons obtained using the proposed method were found to lie within 2*σ* error and agreed with the literature values obtained using TIMS or LA-ICP-MS. However, the ages obtained under dry plasma at 10 *μ*m spot diameter differed from the published data. The results indicated that increasing the spot diameter and introducing 2% water/ethanol vapours into ICP significantly improved the precision, accuracy, and uncertainty at small spot diameter. However, the effect was insignificant at intermediate spot diameter. Furthermore, the effects of spot size and plasma condition on uncertainty, precision, and accuracy strongly depended on the sensitivity of analytes.

## Figures and Tables

**Table 1:** Summary of the operating parameters used for LA-ICP-MS.

GeoLas 2005 laser ablation system	
Wavelength	ArF 193 nm
Pulse length	15 ns
Energy density	6–8 J·cm^−2^
Spot size	10, 16, 24, 32 *μ*m
Repetition rate	3 Hz
Ablation cell gas	Helium (0.5 l·min^−1^)
Makeup gas	Argon (0.631 l·min^−1^ in dry plasma, 0.72 l·min^−1^ in 2% ethanol plasma)
Ablation mode	Single spot

Agilent 7500a ICP-MS	
RF forward power	1380 W
Plasma gas flow rate	14.0 l·min^−1^
Auxiliary gas flow rate	1.0 l·min^−1^
Argon gas flow rate	0.2 l·min^−1^
Aqueous solution uptake rate	0.33 ml·min^−1^ at 0.1 RPS
Skimmer cone	0.4 mm Ni
Sampling cone	1 mm Ni
Ion optical settings	Typical
Dwell time per isotope	6 ms
Detector mode	Dual
Shield torch system	ON
Plasma conditions	Dry plasma or 2% ethanol plasma

**Table 2:** The weighted mean ^206^Pb/^208^U age of SRMs Plešovice, GJ-1, and 91500 under different experimental conditions.

Experimental conditions	Spot size 10 *μ*m				Spot size 16 *μ*m				Spot size 24 *μ*m				Spot size 32 *μ*m				Reference age
	Age	RSD (%)	RE (%)	2*σ*	Age	RSD (%)	RE (%)	2*σ*	Age	RSD (%)	RE (%)	2*σ*	Age	RSD (%)	RE (%)	2*σ*	
	Ma			Ma	Ma			Ma	Ma			Ma	Ma			Ma	
*Plešovice*																	337.1 ± 0.4^a^
Dry plasma	323.7 ± 5.6^*∗*^	3.1	4.0	12.9	328.7 ± 5.9	2.9	2.8	6.1	346.0 ± 2.0	1.4	2.6	4.0	344.9 ± 1.7	1.1	2.8	3.2	338.0 ± 1.0^b^
Ethanol plasma	343.3 ± 4.6	2.8	1.8	8.0	333.8 ± 2.4	1.5	1.0	5.1	339.0 ± 2.4	1.3	0.6	3.3	335.4 ± 2.2	1.1	0.5	2.4	
*GJ-1*																	599.8 ± 4.5^c^
Dry plasma	572.9 ± 7.7^*∗*^	3.2	4.5	17.3	593.8 ± 5.3	1.2	1.0	9.2	596.3 ± 2.8	0.9	0.6	5.9	600.5 ± 3.7	1.0	0.1	5.0	604.6 ± 2.9^d^
Ethanol plasma	597.6 ± 7.8	2.4	0.4	14.8	598.1 ± 4.4	0.7	0.3	8.2	598.6 ± 2.8	0.6	0.2	5.6	597.2 ± 2.1	0.8	0.4	4.6	603.2 ± 2.4^e^
*91500*																	1065.4 ± 0.6^f^
Dry plasma					1057.9 ± 9.7	1.8	0.7	24.8	1060.2 ± 6.1	1.4	0.5	15.5	1058.3 ± 5.8	1.4	0.7	12.2	1061.8 ± 5.3^d^
Ethanol plasma					1061.1 ± 8.0	0.8	0.4	20.3	1064.4 ± 5.7	0.1	0.1	12.8	1064.2 ± 4.3	0.1	0.1	9.7	1064.4 ± 4.8^e^

^*∗*^The concordance was lower than 90% when dry plasma was used at the spot size of 10 *μ*m. ^a^ID-TIMS ^206^Pb/^238^U age of Plešovice from [16]. ^b^LA-ICP-MS ^206^Pb/^238^U age of Plešovice from [17]. ^c^ID-TIMS ^206^Pb/^238^U age (mean of the apparent ages) of GJ-1 from [18]. ^d^LA-ICP-MS ^206^Pb/^238^U age of GJ-1 and 91500 using the U-Pb method from [19]. ^e^LA-ICP-MS ^206^Pb/^238^U age of GJ-1 and 91500 using ablation spot size 20 *μ*m from [20]. ^f^ID-TIMS ^206^Pb/^238^U age of 91500 from [21].

## Data Availability

The data used to support the findings of this study are available from the corresponding author upon request.
